# Haemolysis and lipemia interfere with resistin and myeloperoxidase BioVendor ELISA assays

**DOI:** 10.11613/BM.2019.020703

**Published:** 2019-04-15

**Authors:** Andrea Saracevic, Lora Dukic, Ana-Maria Simundic

**Affiliations:** Department of Medical Laboratory Diagnostics, University Hospital “Sveti Duh”, Zagreb, Croatia

**Keywords:** haemolysis, lipemia, interferences, resistin, myeloperoxidase

## Abstract

**Introduction:**

The aim of our study was to investigate the influence of haemolysis and lipemia on resistin (RES) and myeloperoxidase (MPO) measurement by BioVendor enzyme-linked immunosorbent assays (ELISA).

**Materials and methods:**

Blood was taken from healthy volunteers into lithium heparin tubes. Plasma samples were spiked with Lipofundin® emulsion (B. Braun Melsungen AG, Germany) for lipemia interference testing. Haemolysed samples were obtained by drawing aliquots of heparinized blood through a 26 gauge needle. Index of haemolysis (H), lipemia (L) and triglyceride concentration were measured on Abbott Architect c8000. Haemoglobin concentration was measured on Sysmex XN-1000. Concentrations of RES and MPO in all samples were determined with RES and MPO ELISA kits (BioVendor, Czech Republic). All measurements were performed in triplicate. Biases from the native samples were calculated for both analytes and compared with an arbitrary value (*e.g*. ± 10%).

**Results:**

Triglyceride concentration in the investigated samples ranged from 0.57 to 38.23 mmol/L, which corresponds to L index from - 0.01 to 13.77. Haemoglobin concentration in all samples ranged from 0 to 8 g/L which correspond to H index from 0.05 to 8.77. Both MPO and RES showed significant biases at 1 g/L haemoglobin (58.7% and 66.7%, respectively). Also, both MPO and RES showed significant biases at 4.66 mmol/L triglycerides (33.8% and - 12.2%, respectively).

**Conclusions:**

Resistin BioVendor assays are affected by haemolysis and lipemia already at low degree of interferent. Haemolysis was found to interfere at 1 g/L haemoglobin for both assays, while lipemia interferes at 4.66 mmol/L of triglycerides.

## Introduction

Interferences in immunoassays are highly specific due to numerous analyte dependent type of interferences such as cross-reactions, heterophile, human anti-animal or autoanalyte antibodies, rheumatoid factors, hormone binding and other proteins (*1*, *2*). These types of interferences can be patient specific and are sometimes difficult to recognize especially since immunoassays can produce a diversity of values and can be detected and investigated mostly upon communication with clinicians who receive results that do not fit the clinical picture (*1*, *2*).

The most common endogenous interferences in everyday laboratory practice are haemolysed samples (*3*). Haemolysis occurs due to erythrocyte breakdown and results with release of haemoglobin intracellular proteins, enzymes and cellular debris into the surrounding fluid (*i.e.* serum or plasma). The released components cause various interferences in immunoassay that are method specific and include cross-reactivity with intracellular components, binding of released compounds with the measured analyte or antibodies, enhancing or suppressing the reaction in the detection method or degrading peptide analytes by released proteolytic enzymes resulting in either falsely increased or decreased results (*4*).

Lipemia is not as frequent as haemolysis; nevertheless, it represents a considerable problem in the routine practice. The major causes of lipemia are inadequate time of blood sampling, lipid-containing intravenous infusions and various pathological conditions (diabetes mellitus, pancreatitis, hyperlipidaemia *etc.*) (*5*, *6*).Though lipemia can be avoided in the outpatient units with proper preparation and fasting prior to blood sampling, it is not the case in the intensive care units and emergency departments where parenteral nutrition is sometimes unavoidable and where patients arrive at various time after the last meal, respectively. The most common interference mechanism of lipemia in immunoassays is physical, chemical and spectrophotometric interference and non-homogeneity of the sample, which depends on the measured analyte and method applied (*5*). Additionally, in automated immunoassay, high lipemic content might prevent proper sample aspiration resulting in inadequate volume (*7*).

Unfortunately, evidence has shown that there is a large heterogeneity in reporting interferences in manufacturers’ declarations even for most common chemistry analytes (*8*). It is therefore not so surprising that such reports are scarce or even absent in declarations of new emerging biomarkers.

Myeloperoxidase (MPO) and resistin (RES) are two potentially promising pro-inflammatory biomarkers. Myeloperoxidase is a peroxidase located in the cytoplasmatic granules of leukocytes (neutrophils, lymphocytes, monocytes and macrophages) (*9*). Evidence demonstrates that MPO mediates innate and adaptive immune response and induces the production of proinflammatory cytokines (*10*). Accordingly, MPO was linked to many disease including rheumatoid arthritis, multiple sclerosis, Alzheimer’s and Parkinson’s diseases, cardiovascular diseases (CVD), different types of cancer, autoimmune diseases *etc.* (*4*, *5*). On the other hand, resistin, member of adipocytokines, is expressed mostly in macrophages of the adipose tissue (*11*). Its expression and secretion are up regulated by pro inflammatory stimuli. As a result, increased resistin secretion activates the transcription and thus secretion of numerous proinflammatory cytokines and stimulates the production of superoxide anions that inhibit endothelial nitric oxide synthase resulting in endothelial dysfunction (*12*). Therefore, RES has been implicated in different disease including obesity, insulin resistance, CVD and many others. Many researchers are investigating RES and MPO as potential therapeutic targets and/or prognostic markers and thus, accurate study results are a requirement for making valid conclusions. Therefore, it is important to know which samples are suitable for analysis in order to provide reliable results.

To the best of our knowledge, only one study has investigated the interferences of haemolysis and lipemia on RES, while no study reported these interferences on MPO measurement. In addition, the information regarding haemolysis and lipemia interference on RES and MPO is also missing in the manufacturer’s declaration. Therefore, the aim of our study was to investigate the influence of haemolysis and lipemia on both RES and MPO measurement by BioVendor enzyme-linked immunosorbent assays (ELISA).

## Materials and methods

### Study design

The study was conducted in the Clinical Institute of Chemistry, University Hospital Center “Sestre Milosrdnice” in July 2016. The study was approved by the institutional Ethics Committee and conducted according to the principles of the Helsinki Declaration.

Blood samples were taken from two healthy volunteers after an overnight fast according to the national recommendations for venous blood sampling (*13*). A total of 5 tubes were taken from the first volunteer and 2 tubes from the second participant from the antecubital vein into Vacuette^®^ LH Lithium-heparin (LiH) tube, 4.5 mL, Ref. No. 454049 from Greiner Bio-One (Kremsmunster, Austria) using a Vacuette Multiple Use Draw, 21 gauge needles (Ref. No. 450076) also from Greiner Bio-One. All samples were handled according to manufacturer’s recommendations (*14*). The blood was thoroughly mixed immediately after venipuncture by gently inverting the tubes 5–10 times.

Afterwards, the five LH tubes from the first volunteer were centrifuged at 2000xg for 15 min using the Rotixa 50 RS centrifuge (Hettich Lab Technology, Tuttlingen, Germany). Aliquots of plasma were then spiked with Lipofundin^®^ MCT/LCT 20% emulsion (B. Braun Melsungen AG, Melsungen, Germany) to reach a final volume of 1 mL and different concentrations of Lipofundin^®^, as presented in Table 1.

**Table 1:** Protocol for spiking plasma aliquots with Lipofundin® to achieve lipemic samples

**Sample number**	**Plasma volume (µL)**	**Lipofundin® volume (µL)**	**Lipofundin® concentration (g/L)**
1	1000	0	0
2	995	5	1
3	990	10	2
4	975	25	5
5	950	50	10
6	900	100	20

The blood from the other 2 LH tubes from the second volunteer was divided into 7 aliquots. The aliquots of heparinized whole blood were then drawn through a 26 gauge needle several times in order to achieve different levels of haemolysis. Afterwards, such haemolysed aliquots were centrifuged at 2000xg for 15 min using the Rotixa 50 RS centrifuge to obtain clear plasma without cell debris. The aliquot of heparinized blood intended for the native sample was not drawn through a 26 gauge needle, but was immediately centrifuged at 2000xg for 15 min using the Rotixa 50 RS centrifuge to obtain a clear plasma sample.

Haemolysis (H) and lipemia (L) indices as well as triglyceride concentrations in lipemic samples were measured on Abbott Architect c8000 (Abbott Laboratories, Abbott Park, Illinois, USA). Haemoglobin concentration from the haemolysed samples was measured using the haematology analyser Sysmex XN-1000 (Sysmex, Kobe, Japan). Concentrations of RES and MPO were determined with Human Resistin ELISA and Human MPO ELISA test kits (BioVendor, Brno, Czech Republic) according to the manufacturer’s instructions, each analyte within the same run (*15*, *16*). The intra-assay precisions declared by the manufacturer for RES was 5.2% at 6.34 µg/L and 6.6% at 17.53 µg/L; while the inter-assay precision for was 7.0% at 6.66 µg/L and 8.1% at 23.52 µg/L. The manufacturer did not provide coefficients of variation (CV) for MPO and since there are no available data regarding allowable CV in the literature, we have calculated the mean CV from all of the triplicate MPO measurements and obtained a value of 6.8%.

All measurements were performed in triplicates. The mean value of the three measurements was used for statistical analysis.

### Statistical analysis

The results are presented as mean ± SD. Bias from the concentration in the native sample (B) was calculated for each parameter for all lipemic and haemolytic aliquots, according to the formula: B = (Cx - Cn) / Cn x 100; where Cn represents the mean value of each parameter in the native sample and Cx represents the mean value of each parameter in haemolysed and/or lipemic samples.

Since, there is difficulty in establishing a definitive criteria for both analytes, particularly for resistin because there are no available desirable specifications in the biological variation database, or in the literature, the calculated biases for both analytes were compared with an arbitrary value (*e.g.* ±10%).

## Results

**Figure 1:**
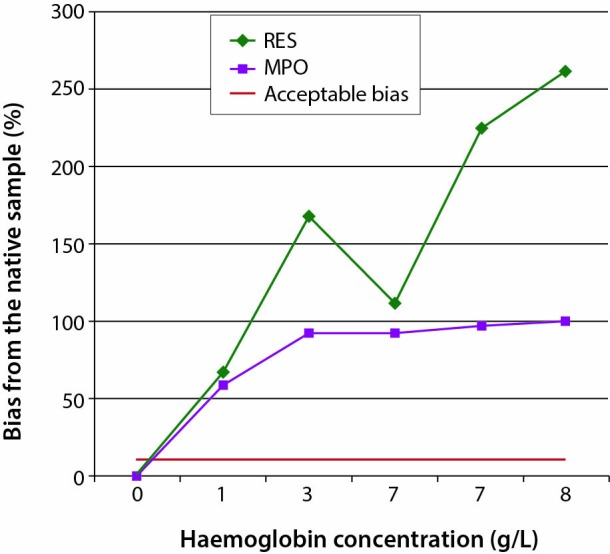
The effect of haemolysis on RES and MPO concentrations. MPO – myeloperoxidase. RES – resistin.

**Figure 2:**
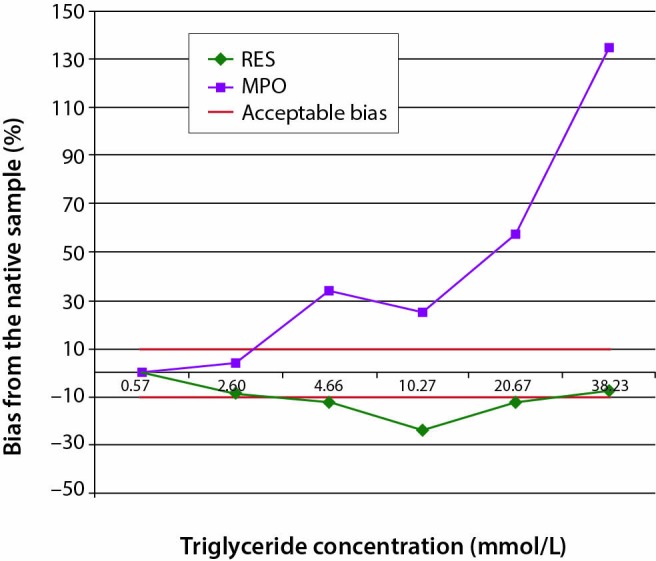
The effect of lipemia on RES and MPO concentrations. MPO – myeloperoxidase. RES – resistin.

The results of the measurements (mean ± SD) carried out in the native, lipemic and haemolysed samples are presented in Tables 2 and 3. Additionally, the biases for RES and MPO are presented as interferograms on Figures 1 and 2.

**Table 2:** The results of the measurements for RES and MPO in native and haemolytic samples

**Sample No.**	**Haemolysis index**	**Haemoglobin (g/L)**	**RES** **(μg/L)**	**RES bias from the native sample (%)**	**Acceptable bias for RES (%)**	**MPO** **(μg/L)**	**MPO bias from the native sample (%)**	**Acceptable bias for MPO (%)**
1	0.05 ± 0.01	0 ± 0	8.9 ± 0.6	/	10.0	164.9 ± 6.9	/	10.0
2	1.34 ± 0.02	1 ± 0	14.8 ± 0.3	66.7		261.7 ± 13.2	58.7	
3	3.37 ± 0.02	3 ± 0	23.9 ± 4.9	168.5		317.1 ± 1.8	92.2	
4	6.97 ± 0.05	7 ± 0	18.7 ± 1.3	110.1		318.0 ± 5.7	92.8	
5	7.79 ± 0.11	7 ± 0	28.9 ± 2.6	224.7		325.3 ± 4.9	97.2	
6	8.77 ± 0.06	8 ± 0	32.2 ± 6.0	261.8		328.3 ± 4.7	99.1	
MPO – myeloperoxidase. RES – resistin.								

**Table 3:** The results of the measurements for MPO and RES in native and lipemic samples

**Sample No.**	**Lipemia index**	**Triglycerides (mmol/L)**	**RES** **(μg/L)**	**RES bias from the native sample (%)**	**Acceptable bias for RES (%)**	**MPO** **(μg/L)**	**MPO bias from the native sample (%)**	**Acceptable bias for MPO (%)**
1	- 0.01 ± 0.00	0.57 ± 0.01	4.6 ± 0.8	/	10.0	46.2 ± 3.8	/	10.0
2	0.59 ± 0.00	2.60 ± 0.01	4.3 ± 0.7	- 7.9		48.2 ± 15.0	4.3	
3	1.28 ± 0.01	4.66 ± 0.03	4.1 ± 0.4	- 12.2		61.8 ± 4.1	33.8	
4	3.28 ± 0.02	10.27 ± 0.15	3.5 ± 0.5	- 23.7		57.8 ± 5.7	25.2	
5	6.57 ± 0.04	20.67 ± 0.25	4.1 ± 0.1	- 12.2		72.8 ± 2.5	57.5	
6	13.77 ± 0.07	38.23 ± 0.29	4.3 ± 0.5	- 7.2		108.3 ± 7.0	134.5	
MPO – myeloperoxidase. RES – resistin.								

## Discussion

The main finding of our study is that haemolysis and lipemia interfere with both MPO and RES BioVendor ELISA measurement. Haemolysis was found to interfere at 1 g/L haemoglobin for both assays, while lipemia interfered already at 4.66 mmol/L triglycerides.

Our results show that for BioVendor ELISA methods, haemolysis causes a positive bias both for MPO and RES, while lipemia causes negative bias for RES and positive bias for MPO up to 38.23 mmol/L triglycerides.

Haemolysis caused positive interference due to the absorbance of haemoglobin at 450 nm, which is the wavelength of the final readings. The level of interference rose as the haemoglobin concentrations were higher. The only discrepancy was evident at 7 g/L of haemoglobin for RES measurements where we obtained lower levels of interference that at the previous point of 3 g/L haemoglobin. A possible reason for such results could be the fact that haemolysis was obtained by drawing whole blood samples through a needle for several times and that there could have been some artefacts within the samples that caused the discrepancy.

Similarly to haemolysis interference, some discrepancies were also found with lipemia testing. For example, for RES, lipemia has shown continuous negative interferences up to 10.27 mmol/L of triglycerides after which the level of interference decreased. A possible explanation could be that Lipofundin® causes non-homogeneity of the sample, but also the fact that the concentration of Lipofundin® was very high which could have caused sample matrix dilution which all together could have contributed to such results.

So far only one study has investigated the influence of haemolysis and lipemia on RES measurement as a part of Vectra^®^ DA, a multi-biomarker test for rheumatoid arthritis activity quantification developed by Crescendo Bioscience (*17*). The authors have spiked serum pool with haemoglobin from Sigma–Aldrich (St. Louis, MO, USA) to reach final haemoglobin concentrations of 0.2 and 2 g/L and with Liposyn from Hospira (LakeForest, IL, USA) to reach lipemic samples with Lyposin concentrations of 2 and 20 g/L. They have calculated the recovery between the samples spiked with haemoglobin/Lyposin and the corresponding controls (samples spiked with equal amount of diluents) and have set arbitrary acceptance criteria for recovery between 80% and 120%. Their results for RES were 98% recovery at haemoglobin 0.2 g/L and 97% at haemoglobin 2 g/L and 107% and 105% for 2 and 20 g/L Lyposin, respectively, thus concluding that haemolysis and lipemia do not interfere with RES measurement, which is somewhat opposite from our findings. There are several possible reasons for such differences. Firstly, the methods used in our study and the one conducted by Eastman and co–workers are not the same. We used ELISA methods while Eastman and colleagues used multiplex sandwich immunoassay electrochemiluminescent technology. Since the methods are produced by different manufacturers, they utilize antibodies that do not target same parts of resistin molecule and have different reaction conditions. It is therefore not surprising that the effect of interference is different with the two immunoassays. Similar discrepancies in interference testing have previously been described for other immunoassays, especially for cardiac troponin, widely used for myocardial infarction diagnosis (*18*). For example, Florkowski and his team investigated the influence of haemolysis on troponin I and T of different manufacturers (Abbott, Ortho Clinical Diagnostics and Roche) and found that very high positive interference for the Ortho Clinical Diagnostics TnI assay and almost no interference for the Abbott Architect TnI. A reason for this discrepancy could be the fact that troponin T in haemolysed sample is cleaved by proteolytic enzymes release by broken red blood cells, which is not the case for troponin I. Additionally, other reports have shown falsely decreased results on Roche fourth and fifth generations and Siemens Vista LOCI assays and low levels of interference for Beckman Coulter AccuTnI+3 assay (*17*). Secondly, we have obtained haemolysed samples by drawing whole blood samples through a 26 gauge needle several times thereby mimicking real-life conditions, while Eastman and colleagues added only haemoglobin into their pools. Since in haemolysed samples, haemoglobin is not the only interferent, but many different enzymes and proteins are also released from neutrophils, it is possible that this fact contributed to such a small level of interference in their experiment.

Unfortunately, literature data regarding the influence of haemolysis and lipemia on MPO measurement is absent. For this reason together with the fact that this analyte is still in the phase of research and has not yet entered clinical practice, even though interference should be evaluated at clinically relevant concentrations, we believe that the investigation on healthy volunteers is a valuable first step in interference testing. In addition, the information regarding these interferences is also missing in the manufacturer’s declaration. BioVendor only provides an advice not to use lipemic or haemolysed samples. Unfortunately, BioVendor is not the only manufacturer with incomplete information in the declarations. There is a large heterogeneity in declarations even within the widely used commercial assays, and there is an ongoing call from the European Federation of Clinical Chemistry and Laboratory Medicine (EFLM) towards the manufacturers in order to provide standardized and transparent declarations about serum indices (*3*). Once this is accomplished, it will be easier for the laboratory professionals to handle such unsuitable specimen.

One possible limitation to our study is a relatively small sample size in which we have investigated the interferences. Additionally, another limitation is the fact that the lowest investigated haemolysis level was at 1 g/L of haemoglobin. This is due to the fact that haemolysis was achieved by drawing whole blood samples through a 26 gauge needle for several times. The level of haemolysis was not always proportional to the numbers of drawing through the needle and therefore we could not control the exact level of haemolysis, which is also the reason why it was very difficult to achieve a low level of haemolysis. It could be possible that lower levels of haemolysis do not cause significant interference and that such samples could be used for analysis. Additionally, haematology analysers might not always be sensitive enough to detect 1 - 8 g/L haemoglobin, which are the concentrations investigated in this study. Free haemoglobin concentrations could also be measured with spectrophotometers; however, since we did not have an available spectrophotometer at the time of the study and therefore we relied on haematology analyser measurements. Nevertheless, since this is the first report regarding haemolysis and lipemia on RES and MPO ELISA measurement we believe it provides evidence based approach to handling haemolysed and lipemic samples and adds valuable contribution to future experiments. Further studies would be advisable, to investigate the effect of interferences on a wider concentration range of RES and MPO. Additionally, we have used Lipofundin® to obtain lipemic samples. Though it does not completely mimic native lipemic samples, such lipid emulsions are still widely used for interference testing. This is mainly because of the fact that such experiments provide valuable information to laboratory professionals and that intensive care units use lipid emulsions for parenteral nutrition and thus they are the cause of a significant proportion of lipemic samples in hospital laboratories (*15*). Finally, we did not exclude that the volunteers had circulating human auto-antibodies or heterophile antibodies in plasma which might explain some unexpected results in our interferograms.

To conclude, as of our results, RES BioVendor assay is affected by haemolysis and lipemia by, already at low degree of interferent. Haemolysis was found to interfere at 1 g/L haemoglobin for both assays, while lipemia interfered already at 4.66 mmol/L triglycerides. In order to obtain reliable results with the investigated ELISA method, such samples should not be used for analysis.
